# A Deep Learning-Based Decision Support Tool for Plant-Parasitic Nematode Management

**DOI:** 10.3390/jimaging9110240

**Published:** 2023-11-06

**Authors:** Top Bahadur Pun, Arjun Neupane, Richard Koech

**Affiliations:** 1 School of Engineering and Technology, Central Queensland University, Rockhampton, QLD 4701, Australia; topbahadur.pun@cqumail.com; 2 School of Health, Medical and Applied Sciences, Central Queensland University, Bundaberg, QLD 4760, Australia; r.koech@cqu.edu.au

**Keywords:** plant-parasitic nematodes, root-knot nematodes, YOLO model, nematode detection/counting, prototype tool, decision support tool

## Abstract

Plant-parasitic nematodes (PPN), especially sedentary endoparasitic nematodes like root-knot nematodes (RKN), pose a significant threat to major crops and vegetables. They are responsible for causing substantial yield losses, leading to economic consequences, and impacting the global food supply. The identification of PPNs and the assessment of their population is a tedious and time-consuming task. This study developed a state-of-the-art deep learning model-based decision support tool to detect and estimate the nematode population. The decision support tool is integrated with the fast inferencing YOLOv5 model and used pretrained nematode weight to detect plant-parasitic nematodes (juveniles) and eggs. The performance of the YOLOv5-640 model at detecting RKN eggs was as follows: precision = 0.992; recall = 0.959; F1-score = 0.975; and mAP = 0.979. YOLOv5-640 was able to detect RKN eggs with an inference time of 3.9 milliseconds, which is faster compared to other detection methods. The deep learning framework was integrated into a user-friendly web application system to build a fast and reliable prototype nematode decision support tool (NemDST). The NemDST facilitates farmers/growers to input image data, assess the nematode population, track the population growths, and recommend immediate actions necessary to control nematode infestation. This tool has the potential for rapid assessment of the nematode population to minimise crop yield losses and enhance financial outcomes.

## 1. Introduction

While the majority of nematodes in the soil are free-living and beneficial to plant growth [1], plant parasitic nematodes (PPN) infest economically important crops worldwide [2]. The damage caused by PPN is estimated to be 215 billion USD globally [3]. These parasites are categorised into two groups: ectoparasitic and endoparasitic. Ectoparasitic nematodes stay outside their host and feed on root tissue using a stylet, whereas endoparasitic nematodes enter the root system and damage root tissue [1]. Sedentary endoparasitic PPNs are most harmful as they inhabit inside of their host plant tissues for the majority of their life span and evolve to feed on root tissue perpetually [1]. The root-knot nematodes (RKN) and cyst nematodes are widely studied pestiferous sedentary endoparasites due to their worldwide agronomic influences [4]. These PPNs use a stylet to penetrate the host plant cell and also release protein to modify the host cell into a nutrient source [5]. On the other hand, plants infested by PPN release molecules that help nematodes move toward the plants and explore feeding sites [6]. The deformation and damage caused by PPN may lead to a malfunction of the root system, making it incapable of absorbing nutrients and water from the rhizosphere. PPN invasion may appear obscure because farmers are often unclear about the symptoms of the microscopic pest infestation [7]. In addition, the symptoms of the affected plants are similar to those caused by nutrient deficiency, poor yield, and wilt appearance [1]. These nematodes can survive through the infestation period of most crops [8,9]. 

To mitigate the problem caused by PPN, farmers commonly use a range of traditional and mechanical methods such as crop rotation, resistant cultivars, and destruction of crop root remnants [10,11]. PPN may also be controlled using chemical nematicides (e.g., organophosphates and carbamates) to optimise crop yield [12]. However, overuse of chemicals may degrade soil quality, become hazardous to the environment [13], and may cause groundwater contamination and poison food materials. Chemical nematicides are thus restricted because of their potentially harmful effects [13]. 

Effective and efficient application of pesticides can eliminate pests while minimising soil damage and environmental contamination, thus maximising crop productivity. This can be facilitated by the accurate identification and quantification of PPN. Traditionally, PPN are identified and counted using a manual microscope. Although this method is relatively simple, it is laborious and time consuming. Alternative approaches to the manual identification and quantification of nematodes include methods based on Deoxyribonucleic Acid (DNA) [14,15], morphological image [16,17], and sequence [18,19]. 

Recently, state-of-the-art deep learning methods have been applied to agricultural problems such as plant phenotyping [20] and fruit detections [21,22]. It has also been used to identify PPN [23]. For instance, a deep learning model was employed to detect and count soybeancyst nematode eggs in the microscopic images [24]. Traditional image processing and computer vision methods are still irreplaceable in domain-specific problems (virtual reality, video processing, and motion captures); however, they have been outperformed by deep learning methods in object detection, image classification, and semantic segmentation [25]. Deep learning and computer vision were used to identify *Globodera pallida* and *Globodera rostochiensis* morphological features (stylet length) [26]. Another deep learning model, NemaNet was developed to detect phytonematodes in soybean crops [27]. The NemaNet model utilised features of the DenseNet and Inception models and achieved an accuracy of 0.9817 and an F1 score of 0.9821. The deep learning model based on ResNet 101 was developed to detect and classify different genera of nematodes [28]. The deep learning models EfficientNetV2B0 ResNet101v2, EfficientNetV2M, and CoAtNet-0 were used to detect nematodes in Indonesian soils [29]. EfficientNetV2M scored the highest accuracy with a 98.66% mean class accuracy and a 98.26% average precision. All these models were only used to detect and classify nematodes. Nevertheless, there remains a gap between model innovation and practical implementation strategies for the benefit of farmers. A significant benefit of pest monitoring and management systems is to collect data and enable farmers to make rapid pest control decisions [30]. 

Thus, this prototype deep learning-based decision support tool (NemDST) was designed to support farmers, offer an easy-to-use interactive interface for image data upload, rapidly detect nematodes, and display estimated nematode populations clearly and efficiently. In addition, this tool monitors nematode population growth and suggests effective nematode control strategies. 

## 2. Related Works

A typical decision support system (DSS) consists of tools to support decision making [31] and often contains some interactive features. With the advancement of information and communication technologies, decision support systems have been widely applied in production and operation management, transportation, logistics, marketing and finance, hospitals, and healthcare facilities [32]. Poor decision making, crop selection, and a shortage of support systems or tools for enhanced crop output are significant hurdles in agricultural production [33]. The essential purpose of a DSS in agriculture is to support farmers in their decision-making processes [34]. A DSS has the potential to facilitate farmers to solve agriculture problems efficiently with a complete understanding of the farm management process. Various decision support systems used in agriculture are shown in Table 1. These decision support systems collect and analyse soil, pest, crop, and environmental data to maximise crop productivity and financial return. Certain DSS include economic data along with optimisation methods to generate comprehensive solutions for the user.

Following our review of the literature, it appears that NemaDecide was the first decision support system developed to manage plant parasitic nematodes by implementing crop rotation with ware and seed potatoes for the succeeding year [35]. The NemaMod component serves as the core engine of NemaDecide, providing nematological information necessary for conducting a cost–benefit analysis on the soil sampling methods. SBN-Watch was used to analyse the effect of crop rotation and sugar beet varieties on the *Heterodera schachtii Schmidt* population [36]. The spatial decision support system was employed to estimate the *Rhagoletis cerasi* severity and reduce the chemical footprint on cherry fruit and its surroundings [37]. This method used two algorithms: one based on day degree mode and the other relying on parameters such as harvest dates, pre-harvest date, and percentage of the trap. The DSS then estimated the efficacy of chemical control strategies. Soil Navigator is another example of a DSS used to investigate soil function and provide soil management guidance to farmers [39]. This system used if-then rules to analyse soil function data such as climate regulation, water purification, primary production, and nutrient cycling. Similarly, a web-based spatial decision support tool was developed to predict soil temperature using generalised additive mixed modeling [40]. 

Few machine learning-based decision support systems have been developed and used in the agricultural sector. A rule-based agriculture DSS incorporated soil, weather, and pest data to support farm management decisions [41]. This DSS enabled farmers to easily test farm management rules for farm production and revenue. Smart irrigation decision support systems use soil sensors to estimate the soil moisture and temperature in addition to weather data such as temperature, rainfall, relative humidity, and dew point [42]. Then, partial least square regression (PLSR) and an adaptive neuro-fuzzy inference system were used to help farmers in irrigation management [42]. Likewise, fuzzy inference system-based IDSS was implemented for irrigation management [43]. The IDSS analysed weather data, soil moisture, and alfalfa height to infer appropriate irrigation time and quantity. Similarly, LDSS was developed to manage agricultural land based on environmental constraints [44]. LDSS analysed soil quality, irrigation data, and ecological factors and computed the optimal land-use structure. In agriculture, decision support systems can be applied for better management of fertiliser, crop rotation, pest monitoring, and optimising livestock diets [47]. AgroDSS uses data mining methods that include predictive modeling, time series clustering functionalities, and structure change detection to assist farmers in effective farm management [38]. The neural network-based DSS was developed to analyse geospatial data and predict seasonal catastrophes and agricultural indicators [45]. Agriculture DSS was also used to manage nutrients and irrigation on the farm [48]. A Bayesian model-based DSS was developed to predict the potential risk of wireworm [46]. The model used soil, field, and weather data to analyse the effects on the wireworm population. This study revealed that the spring season with mild temperatures boosted the wireworm population. Sandy soil is unfavourable to the wireworm development cycle; however, the acidic nature of the soil increases wireworms. Field history with diversified rotation and meadow has a positive effect on the wireworm population. 

### Nematode Management

Once the population of PPN in the sample is determined, their severity is estimated by subtracting the damage threshold, which is defined as the density of the nematode population tolerable based on the particular crops and soil type without causing yield losses [49]. The damage threshold for a particular crop differs for each nematode genus. Damage thresholds are presented in different standards and are expressed in 100 cm^3^ soil and per gram dry root [49]. However, a 200 g sample size was reported to be a reliable and precise measure of the nematode population density [50]. Another study expressed a damage threshold per 250 g of soil [51]. After an accurate assessment of plant parasitic nematodes, a suitable nematode control measure is applied to suppress the nematode population. There are different nematode control methods such as biological control [52], chemical control [53], and cultural practice [54].

The most common control strategy for nematodes involves the utilisation of chemical compounds called nematicides. There are more than 20 chemicals available for chemical control and the most common chemicals are methylbromide and chloropropene [53]. These are fumigant nematicides used before sowing crops and are transferred through the soil in the form of gas. The use of these nematicities, including methyl bromide, are restricted to use because of increasing environmental safety concerns [55]. These products destroy beneficial rhizosphere microorganisms [56] and soil exposed to fumigants is more susceptible to nematode reinfestation [57]. Alternative to fumigants, non-fumigant nematicides are water soluble or formulated in solid. Non-fumigant nematicides such as fluopyram (Velum) and fluensulfone (Nimitiz) are capable of suppressing PPN without damaging the natural ecosystem [58]. Amides, esters, ketones, thioethers, hydrazones, and tioxazafen are the current nematicides used for nematode population suppression [59]. The appropriate dose of nematicide can eliminate 85-90%. However, improperly applied nematicide can delay plant growth and be highly toxic to some plants. 

Crop rotation is one common cultural practice to minimise the nematode population [54]. Crop rotation is accomplished by cultivating highly resistant crops to particular species of nematode. Crop rotation is not applicable to suppress all types of nematode species. Organic materials are used to minimise the RKN population. These organic amendments include cattle manure, chicken litter, and compost [60]. These organic amendments not only suppress the nematode population but also improve plant health and crop yield. Predatory nematodes can be grown from decomposing organic material that feeds plant-parasitic nematodes. Cultivating crop varieties with resistant nematodes is another possible way to minimise plant parasitic nematode infestation. Some genetic varieties of crops have nematode resistance that limits the nematode population growth because of an unsuitable host for nematode reproduction and poor feeding environment [61]. Also, the transgenic plant can decrease the root galls and eggs by suppressing the parasitism gene [62]. These control measures are implemented based on the impact of PPN on crops, seasonal variabilities, and soil properties.

## 3. Materials and Methods

**Figure 1:**
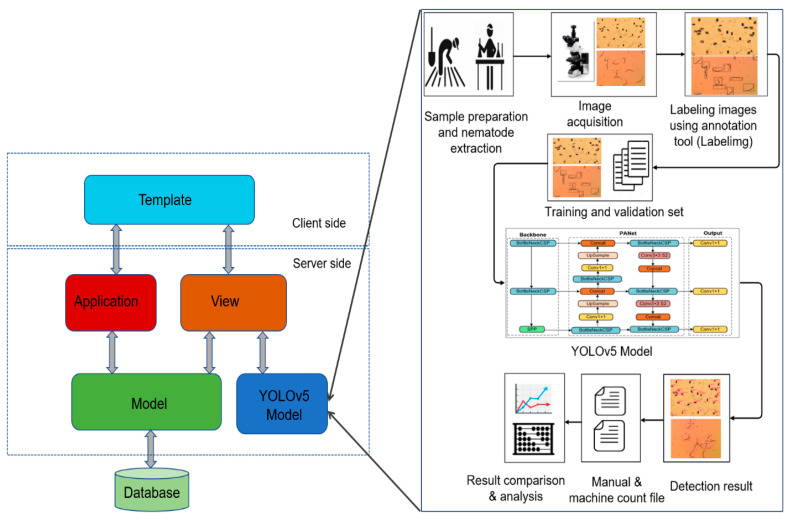
Decision support tool for nematode detection (NemDST).

NemDST was developed in this study using web-based tools integrated with a deep learning model. The deep learning-based pest detection system has three important tasks: data labeling, model training, and model inference [63]. The model was trained using self-collected data; however, the use of publicly available datasets is also possible. The DSS system was designed for PPN and used pre-trained weights to detect and count PPN. NemDST was implemented using Python and Django frameworks as shown in Figure 1. The database management tool is facilitated by MySQL. The PyTorch library was implemented to integrate the YOLO model into the NemDST web system. The YOLO model consists of the pre-trained weight of nematode detection. The libraries used for this decision support system are Django 3.2.5, Pillow 9.5.0, MySQL client 2.1.1, and Torch 2.0.1.

### 3.1. Sample Collection and Nematode Extraction

Initially, the soil was prepared by combining sand and potting mix in a 1:1 ratio. Then, tomato seedlings were transferred to a transparent 600 mL plastic container in a greenhouse located at 24°54′5″ S, 152°18′45″ E (Central Queensland University Science Laboratory, Bundaberg Queensland, Australia). The temperature was maintained at 20 ± 5 °C with 12 h of light. The RKN eggs were inserted into the roots of these plants after five weeks. Subsequently, the roots were investigated five weeks later. The presence of root galls confirmed the infestation of RKN in the plant’s roots.

**Figure 2:**
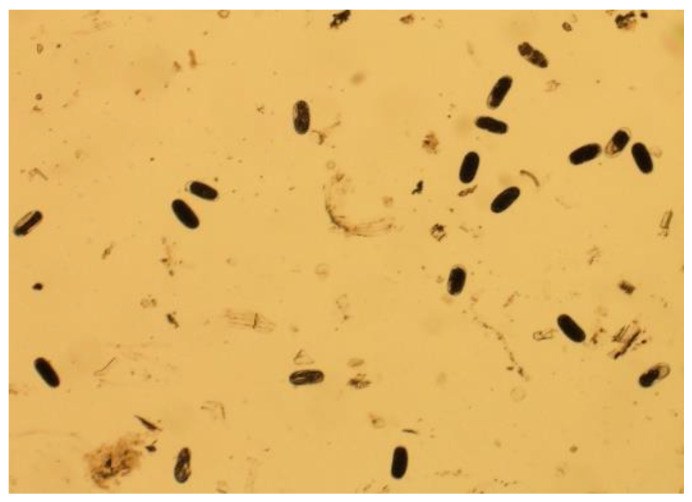
Root-knot nematode (RKN) eggs.

The infected roots were then chopped and washed gently in the lab. This study used Hussey methods to extract nematodes [64]. Roots were cut into 1 cm pieces and poured into a 0.05% concentrated sodium hypochlorite solution. After five minutes, the root particles were washed in a 135 µm aperture sieve stacked with a 25 µm aperture sieve. The eggs and residue were collected in the 25 µm sieve. This leftover was washed out from the sieve using distilled water and kept in the container as a nematode egg sample. This nematode egg sample was put in the petri dish for microscopic inspection. A sample of nematode eggs is shown in Figure 2.

### 3.2. Image Acquisition and Settings

Once the nematode egg sample was ready after extraction, a 5 mL sample was transferred to a 55 mm petri dish and placed on a microscope stage. A BX53 microscopic camera was used with a 4× objective lens to acquire images. The differential interference contrast (DIC) microscopy feature was used with high contrast and manual focus for the optimum image quality. The captured image size was 1200 × 1600 pixels.

### 3.3. Data Collection and Data Preparation

After collecting image data of the nematode eggs, the eggs were labeled separately using the annotation tool (labelImg). The labeling of nematode eggs was manually drawn in the labeling software. The label information of the image was saved in Yolo format in a text file. This nematode dataset was partitioned into training and validation sets in an 80:20 ratio, respectively. Similarly, the RKN egg dataset consisted of 415 images and was split into training and validation sets in an 80:20 ratio. 

### 3.4. YOLO Model

**Figure 3:**
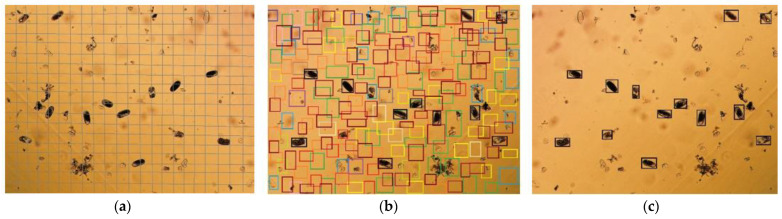
(**a**) S×S grid; (**b**) Bounding Box Prediction; and (**c**) Final Detection.

To detect plant parasitic nematodes, the YOLOv5 model was implemented to discern and count PPN. The YOLO model uses a stage bounding box regressor to formulate object detection problems [65]. The image is divided into sparse grids. The grid cell containing the center of the object is accountable for object detection as shown in Figure 3. The grid cell with a bounding box coordinate is assigned with a probability score, whereas the absence of a bounding box coordinate in the grid cell is denoted with zero scores. YOLO models initiate training by optimising the loss function shown in Equation (1).

(1) $$\mathrm{Loss function}\;=\;\lambda \mathrm{coord}\;\sum_{i=0}^{S^{2}}\sum_{j=0}^{B}1_{ij}^{obj}{\left(x_{i}-{\hat{x}}_{i}\right)}^{2}+{\left(y_{i}-\hat{y}\right)}^{2}]\;+\lambda \mathrm{coord}\;\sum_{i=0}^{S^{2}}\sum_{j=0}^{B}1_{ij}^{obj}{\left(\surd w_{i}-\surd {\hat{w}}_{i}\right)}^{2}+{\left(\surd h_{i}-\surd {\hat{h}}_{i}\right)}^{2}]\;+\sum_{i=0}^{S^{2}}\sum_{j=0}^{B}1_{ij}^{obj}{\left(C_{i}-{\hat{C}}_{i}\right)}^{2}+\lambda_{noobj}\sum_{i=0}^{S^{2}}\sum_{j=0}^{B}1_{ij}^{obj}{\left(C_{i}-{\hat{C}}_{i}\right)}^{2}\;\;+\sum_{i=0}^{S^{2}}1_{i}^{obj}\sum_{c\in classes}{\left(p_{i}\left(c\right)-{\hat{p}}_{i}\left(c\right)\right)}^{2}$$

where $1_{i}^{obj}$ represents the object appearance in cell *i* and $1_{ij}^{obj}$ represents the jth bounding box in cell *i.*

This study employed YOLOv5, YOLOv6, and YOLOv7 to identify RKN eggs. The High-Performance Computing graphic node (NVIDIA ^®^ Tesla^®^ P100, 16 GB Memory, 1328 MHz base clock, 3584 CUDA cores) owned by CQUniversity Australia was used to run these models. These models were trained on three different image sizes (224 × 224, 480 × 480, and 640 × 640) to balance the computation burden associated with high-resolution images and reduced accuracy due to low-resolution images [66]. The parameters were set as 16 batch sizes, 105 epochs, 0.01 learning rate, and 0.937 momentum.

#### 3.4.1. YOLOv5 Model 

**Figure 4:**
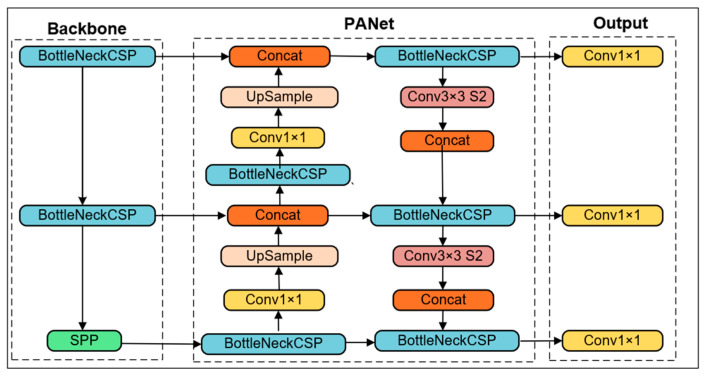
Architecture of YOLOv5 model.

The YOLOv5 model consists of the YOLOv4 head, PANet, and CSPDarknet53 as the main architecture [67] (Figure 4). The YOLOv5 model has five different variants; however, the YOLOv5s model was employed to train on the nematode egg data set [68]. The YOLOv5s model was chosen because it is applicable to lightweight computing. All other variants of the YOLOv5 model were discarded because of a tradeoff between computational burden and speed. 

#### 3.4.2. YOLOv6 Model

**Figure 5:**
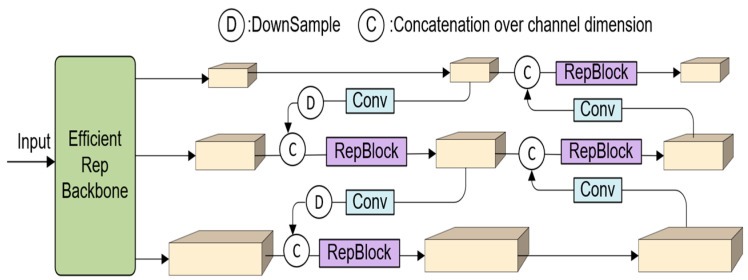
Architecture of YOLOv6.

The YOLOv6 model uses advanced architecture and training strategies to optimise precise localisation and object detection [69] as shown in Figure 5. The model comprises the backbone, neck, and head parts. The backbone consists of a convolutional neural network that is responsible for feature extraction. The neck part uses Rep-PAN to extract deep-level features with high-level attributes. YOLOv6 employs a hybrid channel method in the head that detects objects and classes. 

#### 3.4.3. YOLOv7 Model

**Figure 6:**
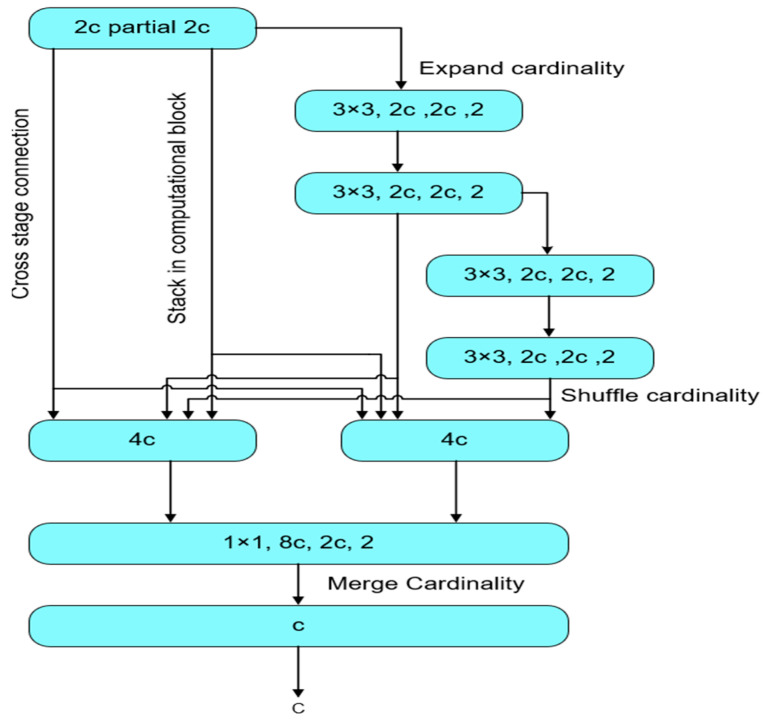
Architecture of YOLOv7.

The YOLOv7 model employs an advanced architecture called an extended efficient layer aggregation network (E-ELAN) with a network learning enhancement feature (Figure 6) [70]. The model also uses a scaling model to achieve greater inference speed. The YOLOv7 model offers planned re-parameterisation training strategies to improve accuracy. The YOLOv7 model is equipped with lead head prediction in the head part to optimise the learning process dynamically and obtain accurate labels of objects. 

### 3.5. Django Web Framework

Django is a Python server-side framework that facilitates Model-View-Controller (MVC) architecture and supports relational databases [71]. The MVC design pattern was utilised to design a web application on which the controller controls user requests and data. The controller can handle multiple views. The view manages presentation logic by rendering appropriate messages and data to the user. Models communicate with databases to manipulate data such as ‘insert’, ‘update’, and ‘delete’. The model also validates integrity constraints and describes the relationship between objects.

### 3.6. Database Diagram

**Figure 7:**
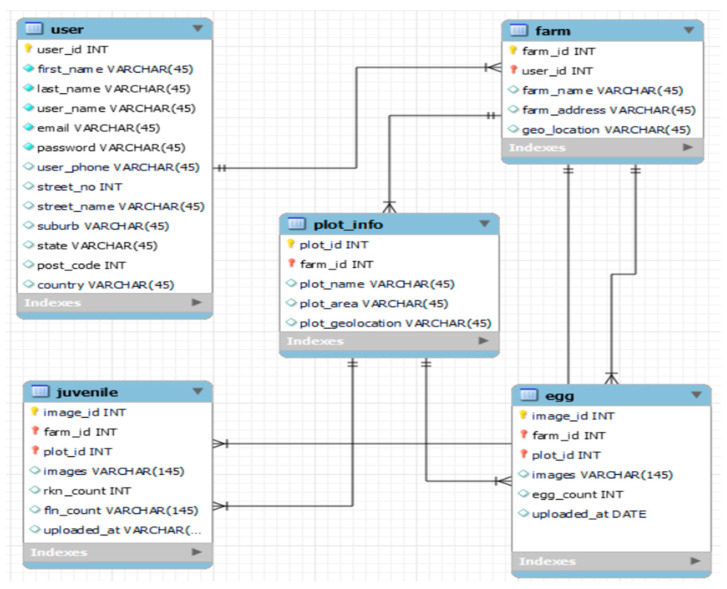
Entity–Relationship diagram of nematodes decision support tool (NemDST).

The MySQL database was used to store NemDST data. Three main modules for the preliminary testing of PPN detection and counting NemDST were designed (Figure 7). The Entity Relationship diagram was designed using the MySQL database tool and consists of five entities. The user entity stores information about the user who can access the nematode decision support system. The farm and the plot info entities store information about the farm. The egg and juvenile entities store information about the image and the number of eggs and juveniles in the image data.

### 3.7. Pest Detection Module 

The pest detection module uses the YOLOv5 model as a background process to classify and count the number of PPN and FLN. For juvenile detection, this module employs the YOLOv5 weight file computed from the RKN detection model [72]. It facilitates the user to choose the type of sample such as eggs, juvenile, and root galls. Then, the user can upload images acquired from a microscopic camera. Once the user submits the images to NemDST, the YOLO module will detect, classify, and count the PPN and FLN and present the totals in the interface. The results of the detection module are shown in the result section.

### 3.8. Evaluation Matrix 

In machine learning, a confusion matrix is an arrangement of actual and predicted classes of objects in rows and columns [73]. The evaluation of classification can be performed in terms of a confusion matrix. The common evaluation measures used in this study are precision, recall, F1 score, mean average precision, coefficient of determination, and mean absolute percentage error. Mean average precision is another popular metric in object detection and classification. To calculate mAP, we first compute the average precision (AP) of each class and compute the mean of AP over all classes as shown in Equation (2).

(2) $$\mathrm{mAP}=\frac{1}{n}\sum_{k=1}^{k=n}AP_{k}$$

where *n* is the number of classes and *AP_k_* is the average precision of class *k*.

The coefficient of determination (Equation (3)) is a dimensionless measurement of the proportion of variance in the dependent variable that can be computed from independent variables [74].

(3) $$R^{2}=1-\frac{\sum_{i=1}^{n}{\left(y_{i}-{\hat{y}}_{i}\right)}^{2}}{\sum_{i=1}^{n}{\left(y_{i}-{\bar{y}}_{i}\right)}^{2}}$$

where *n* denotes the number of samples, *y_i_* represents the actual value, *ŷ_i_* denotes the predicted value, and *ȳ_i_* is the mean value of the actual value for *i* = 1 ….. *n*.

Mean absolute percentage error (Equation (4)) is defined as the proportion of actual values and predicted value to the actual value taken for the n number of data points [75].

(4) $$\mathrm{MAPE}=1/n\;\sum_{1}^{n\;}\frac{\left|y-y\prime \right|}{y}$$

where *y* is the actual value, *y*′ is the predicted value, and *n* is the number of samples.

## 4. Results and Discussion

After training three YOLO models, they are subsequently assessed on the validation set. The result of nematode egg detection on the test set is shown in Table 2. The RKN egg detection result showed that YOLOv7-640 achieved the highest score of precision (0.990), recall (0.994), F1 score (0.996), and mAP(0.991) with an inference time of 15.1 milliseconds (ms). However, YOLOv5-640 attained similar accuracy while retaining an inference time of 3.9 ms. Although YOLOv7-224 showed a 3.5 ms inference time, it produced lower accuracy, demonstrating a precision of 0.955, recall of 0.993, F1 score of 0.973, and mAP of 0.987, respectively. The inference time was lowest (2.5 ms) on YOLOv5-224 detection whereas the highest was on YOLOv5-640 (3.9 ms). In addition, the YOLOv6 model with a 224 × 224 input image found the lowest accuracy with a precision of 0.913, recall of 0.940, F1 score of 0.927, mAP of 0.970, and a 3.01 ms inference time. The RKN egg detection results showed a slight reduction in the performance with low-resolution images. All the models demonstrated more than a 90% accuracy; nevertheless, the YOLOv6 model had slightly lower accuracy compared to YOLOv5 and YOLOv7. The YOLOv5-640 model exceeded the results obtained from the convolutional selective autoencoder (CSAE) developed to detect and count soybean-cyst nematode eggs [24]. In addition, the YOLOv5 model detected nematode eggs faster compared to the CSAE model which required 1 frame per second. Nevertheless, the deep learning model performance decreases as target domains are shifted because it is sampled on the same distribution. To avoid these issues, various deep domain adaptation techniques can be implemented, such as the feature-based adaptation method, instance-based adaptation, feature reconstruction, feature transformation, adversarial method, and discrepancy-based method [76]. 

**Figure 8:**
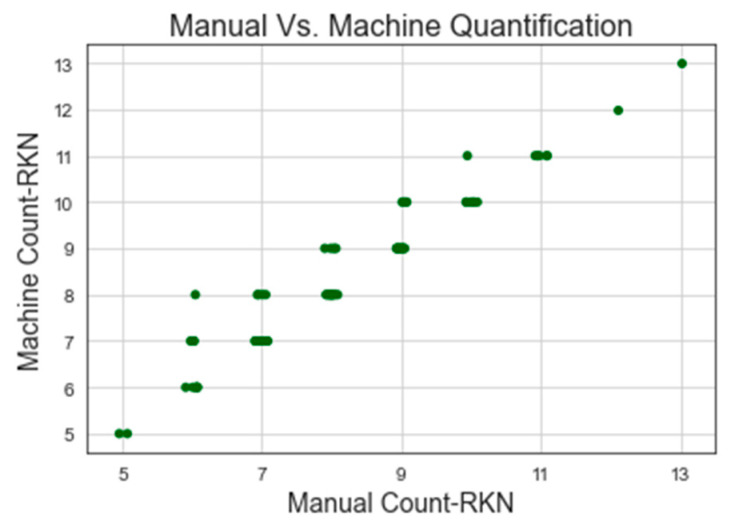
Manual and machine counting of RKN egg using YOLOv7-640.

**Figure 9:**
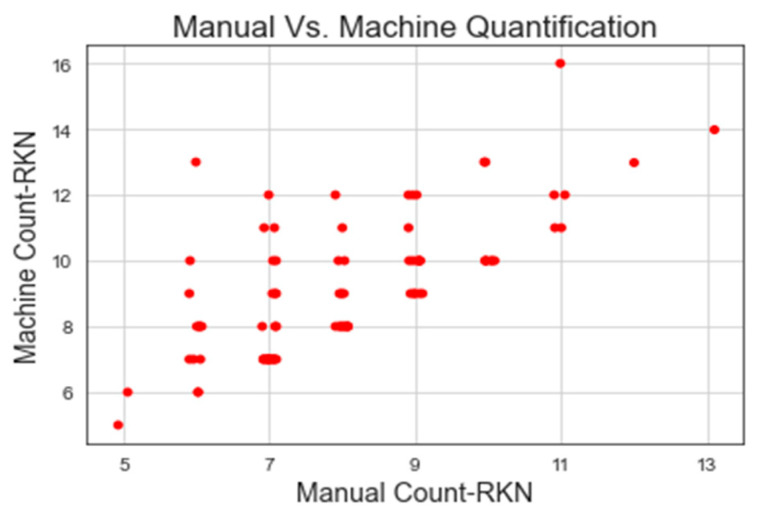
Manual and machine counting of RKN egg using YOLOv6-480.

The machine and manual counting results of RKN eggs are shown in Table 3 and Figure 8 and Figure 9. YOLOv7-640 attained the highest correlation of manual and machine counting of RKN eggs with R^2^ = 0.964 and MAPE = 3.582, whereas YOLOv6-480 showed the lowest correlation of manual and machine counting with R^2^ = 0.957 and MAPE = 2.978. The lowest correlation was due to an increase in the number of false positives, as the YOLOv6 model detected soil particles as RKN eggs. This might be caused by insufficient training data. The YOLOv7-640 surpassed the outcome of RKN egg detection using conventional image processing and computer vision methods [77]. YOLOv7-640 achieved results similar to those of counting soybeancyst nematode eggs using CSAE [78]. The threshold method used in the conventional image processing method cannot differentiate soil particles that have a length and width similar to the RKN eggs [77]. YOLOv5-640 and YOLOv7-640 outperformed the image processing method used in [77] and accurately discriminate between soil particles and nematode eggs. However, the accuracies of both YOLOv5-640 and YOLOv7-640 could not attain the top scores because they were unable to discriminate between certain soil particles that share the same colour as RKN eggs. Also, the model was unable to distinguish spoiled RKN eggs, which seemed slightly faded and transparent compared to RKN fertile eggs. This problem might be avoided by using a training model with a large variety of data (limited amount of data was used to train the model in our study).

Most nematode studies are limited to the identification task [26,27,29]; nonetheless, this study explored the detection and counting of RKN eggs which is essential in estimating the magnitude of the negative impact on plants or animals. Although C. elegans can be cultured in the lab, economically important plant-parasitic nematodes are dependent on the feeding of host plant tissue and cannot be cultured in substantial numbers [7,79,80]. Thus, it makes them challenging pathogens for laboratory experiments. The characteristics of image data rely on the extraction methods used for nematodes. This study employed a root extraction method to collect samples that can be further expanded using the soil extraction method. As nematode sample images are acquired from the camera attached to the specific microscope, these sample images can be taken from a variety of sensors and microscopes to efficiently obtain high-quality image data.

Even though YOLOv5-640 achieved the highest accuracy in terms of detecting nematodes, this model can be modified in terms of the loss function and optimisation criteria to attain greater accuracy in nematode detection and counting. In addition, the YOLOv5-640 model can be compared to several other convolution neural networks for nematode detection. Further, these models can be utilised to assess different datasets of nematodes from a wider range of geographical locations and growing conditions or even detect microorganisms and pests. In addition, this study can be further explored by utilising the latest YOLOv8 model to estimate the nematode population.

### Simulation Result of YOLOv5-Based DST

**Figure 10:**
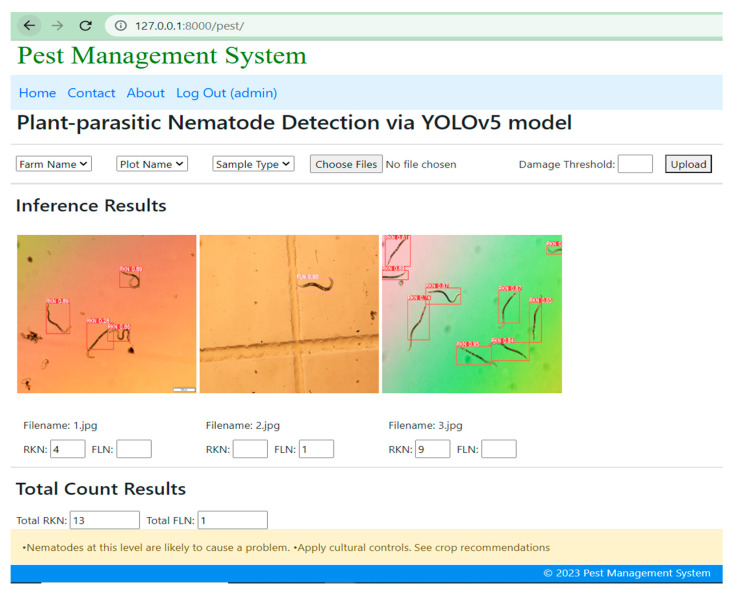
Detection and counting of root-knot nematode (RKN) juveniles.

**Figure 11:**
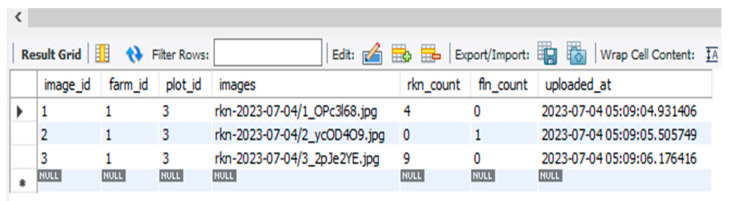
Root-knot nematode detection result saved in database table (juvenile).

**Figure 12:**
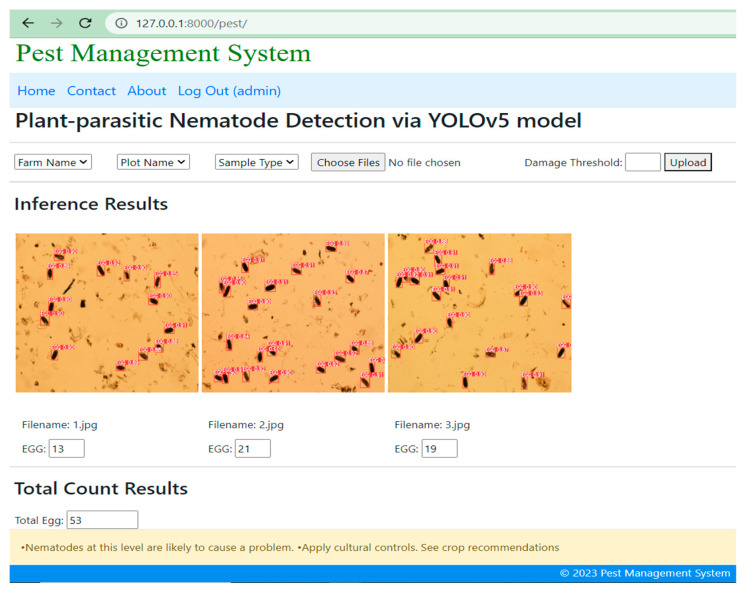
Detection and counting of root-knot nematode eggs.

**Figure 13:**
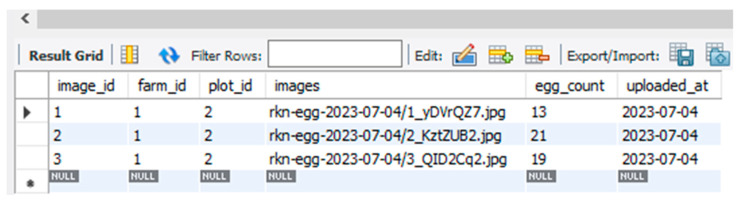
Root-knot nematode egg detection results saved in the database table (egg).

Once the user submits the plant-parasitic nematode images, the DST system displays the detection of nematodes and their quantity as shown in Figure 10. The inference speed for nematode detection was four frames per second whereas the inference speed for nematode egg detection was 1.3 frames per second in normal computing resources. The detection and counting of RKN and FLN nematodes and the counting results are saved in the juvenile table of the MySQL database (Figure 11). Once the user submits the image of the egg sample, the DST displays the inference and counting results as shown in Figure 12, and the detection and counting results are saved in the egg table of the MySQL database (Figure 13).

This study explored a deep learning-based decision support tool for plant-parasitic nematode management that holds promising applications in modern agriculture. By implementing state-of-the-art deep learning techniques and integrating them with the YOLOv5 model, this tool offers a great advantage in rapidly estimating nematode populations, particularly their juveniles and eggs. The performance of the YOLOv5 model exhibited significant application in pest management. This tool enables farmers to assess nematode populations efficiently, enabling timely intervention and minimising crop yield losses. The implications of this innovation extend beyond improved yields; it has the potential to enhance financial outcomes, ensure food security, and reduce the environmental impact caused by unnecessary pesticide use. In comparison to conventional machine learning methods like random forest [81,82], support vector machines [83], and discriminant analysis [84], the deep learning-based approach extracts information from complex image data, and thus, it is appropriate for overcoming the challenges posed by plant-parasitic nematodes and pest management.

When compared with similar studies in the context of plant diseases in tropical crops, this research has significant applications. Plant diseases can have catastrophic effects on tropical crops, where climatic conditions often favour the proliferation of pests and diseases [82,85]. While other machine learning methods have demonstrated utility in disease detection, their efficacy can be limited in the face of nuanced and rapidly evolving pathogens [86,87]. The deep learning-based decision support tool discussed in this study showcases adaptability and versatility by effectively solving nematode adversity and the prevalent issue of pests in agriculture [88]. This tool can handle image-based data and offers a path toward addressing plant diseases as well, potentially revolutionizing disease management in tropical crops. The ability to harness cutting-edge technology for pest and disease management could pave the way for more sustainable and resilient agricultural practices, ensuring a stable food supply for regions heavily reliant on crops.

## 5. Conclusions

This study built a new prototype decision support tool (NemDST) integrated with a deep learning-based detection model that facilitates rapid and innovative methods for plant parasitic nematode identification and assesses the magnitude of nematode infestation. The NemDST utilises the state-of-the-art YOLOv5 to classify and count plant parasitic nematodes at juvenile and egg stages. The system provides fast and efficient tools to estimate the plant-parasitic nematode population and provide information about nematode management tactics. Further, the NemDST stores nematode data in the database, helps the farmer keep track of the nematode population for future studies, and applies appropriate nematode management practices. The NemDST can be further extended to develop integrated pest management (IPM) using machine learning and deep learning technologies.

## Figures and Tables

**Table 1:** Decision support systems used in agriculture.

DSS	Application	Methods	Reference
NemaDecide	Crop rotation	Stochastic and probabilistic methods	[35]
SBN-Watch	Crop rotation	Seinhorst equations	[36]
Spatial DSS	Pest management	Degree day model and harvest date estimation	[37]
AgroDSS	Prediction of pest population	Random forest and time series analysis	[38]
Soil Navigator	Soil function assessment and management	Decision Tree, if then rules	[39]
Spatial DST	Soil temperature prediction	Generalised additive mixed model	[40]
Great Plains Framework for Agricultureal Resource Management (GPFARM)	Weed control, fertilisation, and harvest management	If then rules	[41]
Smart Irrigation Decision Support System (SIDSS)	Irrigation management	Partial least square regression and fuzzy inference system	[42]
Irrigation decision support system (IDSS)	Irrigation management	Fuzzy inference system	[43]
Land-use Decision Support System (LDSS)	Land Management	Multivariate linear programming	[44]
Deep neural network-based DSS	Crop yield prediction	Back propagation neural network and grey decision-making system	[45]
Bayesian model-based DSS	Wireworm pest risk assessment	Bayesian model	[46]

**Table 2:** Result of nematode detection using YOLOv5, YOLOv6, and YOLOv7.

Model	Precision	Recall	F1-Score	mAP (IoU Threshold 50%)	Inference Time
YOLOv5-224	0.951	0.896	0.924	0.917	2.5 ms
YOLOv5-480	0.974	0.991	0.983	0.993	2.7 ms
YOLOv5-640	0.992	0.959	0.975	0.979	3.9 ms
YOLOv6-224	0.913	0.940	0.927	0.970	3.01 ms
YOLOv6-480	0.962	0.970	0.966	0.983	5.15 ms
YOLOv6-640	0.969	0.960	0.965	0.982	8.22 ms
YOLOv7-224	0.955	0.993	0.973	0.987	3.5 ms
YOLOv7-480	0.991	0.990	0.990	0.995	8.3 ms
YOLOv7-640	0.990	0.994	0.996	0.991	15.1 ms

**Table 3:** Correlation between manual and machine nematode egg detection using YOLOv5, YOLOv6, and YOLOv7.

Model	Coefficient of Determination (R^2^)	Mean Absolute Percentage Error (MAPE)
YOLOv5-224	0.850	12.311
YOLOv5-480	0.944	4.375
YOLOv5-640	0.957	2.978
YOLOv6-224	0.763	14.191
YOLOv6-480	0.726	16.243
YOLOv6-640	0.775	16.458
YOLOv7-224	0.846	12.119
YOLOv7-480	0.940	4.011
YOLOv7-640	0.964	3.582

## Data Availability

The data used in this study can be accessible from corresponding author (CA) upon reasonable request.
